# Down-regulatory mechanism of mammea E/BB from *Mammea siamensis* seed extract on Wilms’ Tumor 1 expression in K562 cells

**DOI:** 10.1186/s12906-016-1107-z

**Published:** 2016-05-18

**Authors:** Methee Rungrojsakul, Trinnakorn Katekunlaphan, Aroonchai Saiai, Chadarat Ampasavate, Siriporn Okonogi, Colleen A. Sweeney, Songyot Anuchapreeda

**Affiliations:** Department of Pharmaceutical Sciences, Faculty of Pharmacy, Chiang Mai University, Chiang Mai, 50200 Thailand; Department of Chemistry, Faculty of Science, Chandrakasem Rajabhat University, Bangkok, 10900 Thailand; Department of Chemistry, Faculty of Science, Chiang Mai University, Chiang Mai, 50200 Thailand; Department of Biochemistry and Molecular Medicine, UC Davis School of Medicine, Sacramento, CA 95817 USA; Department of Medical Technology, Faculty of Associated Medical Sciences, Chiang Mai University, Chiang Mai, 50200 Thailand; UC Davis Cancer Center, Research Building III, Room 1100A, 4645 2nd Avenue, Sacramento, CA 95817 USA; Division of Clinical Microscopy, Department of Medical Technology, Faculty of Associated Medical Sciences, Chiang Mai University, Chiang Mai, 50200 Thailand

**Keywords:** Mammea E/BB, *Mammea siamensis*, WT1, AP-1, ERK1/2, Cell cycle arrest, K562 cells

## Abstract

**Background:**

Wilms’ tumor 1 (WT1) is a biological marker for predicting leukemia progression. In this study, mammea E/BB, an active compound from Saraphi (*Mammea siamensis*) seed extract was examined for its effect on down-regulatory mechanism of *WT1* gene expression, WT1 protein and mRNA stability, and cell proliferation in K562 cell line.

**Methods:**

*M. siamensis* seeds were obtained from the region of Chiang Mai (North of Thailand). Mammea E/BB was extracted from seeds of *M. siamensis*. WT1 protein expression and stability were evaluated by Western blot analysis. WT1 mRNA stability was assessed by qRT-PCR. WT1-DNA binding and WT1 promoter activity were assayed by ChIP assay and luciferase-reporter assay, respectively. Cell cycle arrest was studied by flow cytometry.

**Results:**

Treatment with mammea E/BB led to down-regulation of WT1 expression. The suppression of WT1 expression did not involve protein and mRNA degradation. Rather, WT1 protein was down-regulated through disruption of transcriptional auto-regulation of the *WT1* gene. Mammea E/BB inhibited WT1-DNA binding at the WT1 promoter and decreased luciferase activity. It also disrupted c-Fos/AP-1 binding to the WT1 promoter via ERK1/2 signaling pathway and induced S phase cell cycle arrest in K562 cells.

**Conclusion:**

Mammea E/BB had pleotropic effects on kinase signaling pathways, resulting in inhibition of leukemia cell proliferation.

**Electronic supplementary material:**

The online version of this article (doi:10.1186/s12906-016-1107-z) contains supplementary material, which is available to authorized users.

## Background

**Fig. 1:**
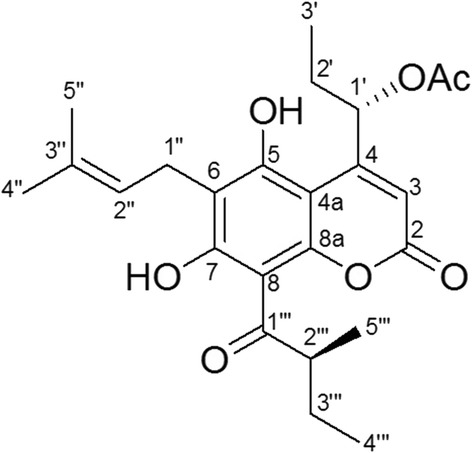
Chemical structure of Mammea E/BB

*Mammea siamensis* (Miq.) T. Anders is a Thai medicinal plant belonging to the family of Guttiferae, and is known in Thai as “Saraphi” [1]. Previous phytochemical studies of *Mammea* have led to the isolation and structural determination of coumarins (mammea, surangin, therapin, calanone, mammeanoyl, etc.) found in the root, leaf, twig, stem, bark, flower, and seed of *M. siamensis*, *M. harmandii*, *M. americana*, *M. longifolia*, and other species of *Mesua* and *Calophyllum* [2–10]. Coumarins are well-known natural products that have been shown to have various biological activities, such as insecticidal [11], antioxidant [5, 12, 13], antibacterial [5], antifungal [14], anti-malarial [15], anti-HIV [16], and anticancer activities [4, 7, 10, 12, 13]. A previous study reported the isolation and structural determination of phenolic compounds from *M. siamensis* seeds, including siamensone A, surangin B, mammea E/BB (Fig. 1), and δ-tocotrienol [6]. Recently, compounds from the flowers of *M. siamensis* were found to exert antiproliferative actions through apoptotic cell death in leukemia cells [10].

The *Wilms’ tumor 1* (*WT1*) gene is located at chromosome 11p13 and encodes a 48–57 kDa WT1 protein that functions as a DNA-binding transcription factor involved in growth regulation, and is necessary for the induction of cell differentiation [17]. WT1 has been purported to have both oncogenic and tumor suppressor functions. Low levels of WT1 protein expressions have been found in normal blood cells, while high levels of WT1 protein have been found in leukemia patients [18]. Previously, pure curcumin was shown to repress *WT1* gene expression in both primary and leukemic cells [19]. In addition, Semsri et al. reported that pure turmeric curcumin affected WT1 protein-promoter binding and decreased WT1 mRNA and protein levels through inhibition of the PI_3_K/PKC_α_/JNK pathway in K562 cells [20]. Moreover, expression of the *WT1* gene and its product has been used as biological markers for diagnosis and evaluation of the prognosis in leukemia and minimal residual disease (MRD) [18, 21]. A previous study revealed that mammea E/BB also suppressed WT1 protein expression when compared to surangin A and surangin C [22]. However, the down-regulatory mechanism was unknown. The current study therefore aimed to examine the inhibitory mechanism of mammea E/BB on *WT1* gene expression, WT1 protein and mRNA stability, and cell proliferation in K562 cell line.

## Methods

### Materials

*M. siamensis* seeds were collected from Chiang Mai University, Amphoe Muang, Chiang Mai province, Thailand in May 2010. The plant material used in this study was identified by Mr. James Franklin Maxwell. A voucher specimen (J.F. Maxwell, No.92-70) is deposited in the CMU herbarium, Faculty of Science, Chiang Mai University, Chiang Mai, Thailand. RPMI-1640, fetal bovine serum, *L*-glutamine, penicillin/streptomycin, Quick-Change Site Mutagenesis Kit, and HRP-conjugated goat anti-rabbit IgG were purchased from Invitrogen™ Life (Carlsbad, CA, USA). MTT dye and StaphA cells were purchased from Sigma-Aldrich (St Louis, MO, USA). Trypan blue dye solution was purchased from AMRESCO^®^ (Solon, OH, USA). Rabbit anti-WT1 and rabbit anti-GAPDH was purchased from Santa Cruz Biotechnology (CA, USA). Rabbit anti-p-ERK1/2 was purchased from New England BioLabs (Beverly, MA, USA). Mouse anti-cyclin B and Mouse anti-cyclin A were purchased from BD Transduction Laboratories™ (USA). Mouse anti-CDK1 (cdc2) was purchased from Abcam^®^ (MA, USA). Rabbit anti-c-Fos and PathScan^®^ Intracellular Signaling Array Kit (Chemiluminescent Readout) were purchased from Cell Signaling Technology^®^ (Danvers, MA, USA). HRP-conjugated anti-mouse IgG, Go Taq^®^ DNA Polymerase, Dual-Luciferase Reporter Assay Kit, and β-galactosidase Assay Kit were purchased from Promega (WI, USA). Enhanced chemiluminescence detection kit and Dream Taq Green PCR master mix were purchased from Thermo Scientific (Miami, USA). E.Z.N.A.^®^ Total RNA Kit I was purchased from Omega bio-tek (Norcross, GA, USA). RevertAid First Strand cDNA Synthesis Kit was purchased from Thermo scientific (EU). Cycloheximide (CHX) and actinomycin D were purchased from Calbiochem (USA). Qiaquick PCR kit was purchased from Qiagen (CA, USA).

### General experimental procedures

Optical rotations were measured with a JASCO P-1020 polarimeter. 1D and 2D NMR spectra were recorded using a Bruker AVANCE 400 NMR spectrometer. Chemical shifts (δ) are expressed in ppm with reference to the solvent signals. UV spectra were measured using a SHIMADZU UV-2450 spectrophotometer.

### Extraction and isolation of mammea E/BB

Mammea E/BB was purified from seeds of *M. siamensis* using column chromatography, extraction, and isolation as previously described [22]. Mammea E/BB was obtained as a pale yellowish gum with [α]_D_^27^ −65.7° (c = 0.40, MeOH). The UV spectra of mammea E/BB exhibited absorption maxima bands at 337 and 265 nm; these are characteristic for coumarin [23]. The absolute stereochemistry at C-1’ and C-2′′′ was assigned to be *S* from its negative optical rotation value [12]. The mammea E/BB identity was confirmed by comparison of the ^1^H and ^13^C NMR spectra data (Additional file 1: Figure S1 and Additional file 2: Figure S2) with those reported in the literature [24, 25].

### Cells and cell culture conditions

The K562 cell line, a model of WT1-overexpressing leukemic cells, was cultured in RPMI-1640 medium supplemented with 10 % fetal bovine serum, 1 mM *L*-glutamine, 100 units/ml penicillin, and 100 μg/ml streptomycin, and incubated under 95 % relative humidity with 10 % CO_2_ at 37 °C.

### MTT assay

The cytotoxicity of mammea E/BB was evaluated using the MTT assay. Briefly, K562 cells (1.0 × 10^4^ cells/well) were cultured in 96 well plates containing 100 μl medium prior to treatment for 24 h. After that, 100 μl of fresh medium containing various concentrations (0–116 μM) of the test compound were added to each well and incubated for 72 h. The MTT dye solution was added and the percent cell viability, IC_50_, and IC_20_ values were calculated as previously described [22].

### Trypan blue exclusion assay

Cell proliferation was measured by the trypan blue exclusion method. Cells were treated with various concentrations of mammea E/BB for 72 h. Then cells and 0.4 % trypan blue dye were mixed and counted using a light microscrope. All experiments were performed in triplicate.

### Protein extraction and Western blotting

K562 cells were treated with mammea E/BB for 72 h, after which the cells were collected, washed twice with cold PBS, and lysed with cold RIPA buffer (50 mM Tris-HCl, 150 mM NaCl, 1 % Triton X-100, 0.5 mM EDTA, 0.1 % SDS, and 0.001 % protease inhibitor cocktail) for whole protein extraction. Whole protein lysates (25 μg) were loaded onto 12 % SDS-PAGE and then transferred to PVDF membranes. Membranes were blocked with 5 % skim milk and probed with rabbit anti-WT1, rabbit anti-p-ERK1/2, mouse anti-cyclin B, mouse anti-cyclin A, and mouse anti-CDK1 (cdc2) at 1:1,000 dilution. Rabbit anti-GAPDH at a dilution of 1:1,000 was used for protein loading control. The reaction was followed by HRP-conjugated goat anti-rabbit IgG at 1:10,000 dilution and HRP-conjugated anti-mouse IgG at 1:10,000 dilution. Proteins were visualized using an enhanced chemiluminescence detection kit and the chemiluminescent signals detected using an Alpha Innotech gel imaging system (Cell Biosciences Inc., CA, USA). Densitometry was performed using Alpha Innotech software. The band density of the loading control was used to normalize the band densities of proteins of interest to obtain the relative normalized expression level as compared to the exposed control.

### RNA isolation and reverse transcription PCR analysis

K562 cells were treated with mammea E/BB and the cell pellets were collected. The pellets were washed twice with cold PBS and isolated by E.Z.N.A.^®^ Total RNA Kit I according to the manufacture’s protocol. The cDNA (1 μg) was synthesized from total RNAs using a RevertAid First Strand cDNA Synthesis Kit with random hexamer primers according to the manufacture’s instructions. RT-PCR analysis was carried out with a Mastercycler^®^ personal (Eppendorf, CA, USA) instrument with Dream Taq Green PCR master mix probe-based chemistry for WT1 and GAPDH.

### Protein degradation and mRNA half-Life analysis

K562 cells were incubated (0, 3, 6, 12, and 24 h) with or without 5 μM MG132 (proteasome inhibitor) and confirmed by 50 μg/ml cycloheximide (CHX) in the presence or absence of 3.5 μM of mammea E/BB. The WT1 mRNA stability of K562 cells was assessed with 4 μM actinomycin D in the presence or absence of 3.5 μM mammea E/BB.

### Chromatin immunoprecipitation (ChIP) assay

The concept of chromatin immunoprecipitation or ChIP is an experimental form of immunoprecipitation used to investigate the interaction between proteins and DNA in the cell [26]. Briefly, cells were treated with 3.5 μM mammea E/BB for 72 h then crosslinked with 1 % formaldehyde for 10 min, followed by addition of 0.125 M glycine to stop the crosslinking. Cells were suspended in cell lysis buffer (5 mM PIPE, 85 mM KCl, 1 % NP40, and protease inhibitors), incubated on ice for 15 min and centrifuged at 5,000 rpm at 4 °C for 5 min. The nuclei pellet was resuspended in lysis buffer (50 mM Tris-HCl, pH 8.0, 10 mM EDTA, 1 % SDS, and protease inhibitors) and incubated on ice for 10 min before sonication. The chromatin lysate was precleared with StaphA cells before immunoprecipitation at 4 °C overnight. Antibodies included WT1 (C-19), c-Fos, and normal rabbit IgG as a negative control. On the following day Staph A cells were added to precipitate the IgG/protein/DNA complex. The StaphA cell pellets were washed extensively, followed by elution and reversal of protein-DNA crosslinking. The protein was removed from the DNA lysate using the Qiaquick PCR kit. Standard PCR was performed on the precipitated DNA template using Go Taq^®^ DNA Polymerase.

### Primer design

The human WT1 promoter sequence was obtained from NCBI, accession # U77682. Primer design for the WT1 promoter region utilized Primer3 Input (version 0.4.0), UCSC Genome Browser and Vector NTI advance 10: WT1 promoter primer sequence No.1 consisted of the forward primer (CTGAACGGACTCTCCAGTG) and reverse primer (CGCTGCCTTGAACTCCTTAC); WT1 promoter primer sequence No.2 consisted of the forward primer (GGCCCCTCTTATTTGAGCTT) and reverse primer (CAAGAGGAA GTCCAGGATCG).

### Promoter luciferase-reporter assay

WT1 promoter vectors were a kind gift from Professor Dr. Takashi Murate (Department of Medical Technology, Nagoya University Graduate School of Heath Sciences, Japan). The WT1 promoter sequence, including the WT1 and c-Fos/AP-1 binding site, and 301 bp reporter constructs was inserted into the pGL3 basic vector. For the mutant pGL3 construct, core nucleotides of a potential WT1 binding site were altered using a Quick-Change Site Mutagenesis Kit with PCR primers for the WT1 consensus sequence located at -50 to-39; GTGTGGGAGCC [27] was mutated to *A*T*A*TG*AT*A*T*C*A*. K562 cells were co-transfected with the construct vector and β-galactosidase (β-Gal) for 24 h and then treated with mammea E/BB. Co-transfected cells were lysed with lysis buffer. Luciferase activity of the lysis solution was measured using a Dual-Luciferase Reporter Assay Kit, β-galactosidase Assay Kit and a GloMax 96 Microplate Luminometer with dual injections (Promega, USA).

### Intracellular signaling array

The intracellular signaling pathway involved in leukemic cell growth inhibition after treatment was determined using a PathScan^®^ Intracellular Signaling Array Kit (Chemiluminescent Readout).

### Cell cycle analysis

Cell cycle analysis was done via flow cytometry using propidium iodide (PI), which represents the content of nuclear DNA. K562 cells (1 × 10^5^ cells/ml) were cultured in complete RPMI-1640 medium with or without mammea E/BB for 72 h. After treatment, cells were washed twice with ice-cold PBS and prepared as a single-cell suspension. Then, the cells were fixed with ice-cold 70 % absolute ethanol for 30 min and harvested by centrifugation. The cell pellets were then washed with ice-cold PBS and stained with PI solution (0.1 % triton X-100, 8 μg/ml RNase A, 2 mM EDTA, and 20 μg/ml PI) in the dark at 4 °C. Thereafter, the red fluorescence was measured on a BD FACSCaibur flow cytometer (Becton Dickinson, San Jose, CA, USA). A minimum of 50,000 events was collected per sample. The data were analyzed using FlowJo 7.6.5 software.

### Statistical analysis

All the data were expressed as the mean ± standard deviation (SD) or mean ± standard error of mean (SEM) from triplicate samples of three independent experiments. The statistical differences between the means were determined using one-way ANOVA. The differences were considered significant when the probability value obtained was found to be less than 0.05 (*p* < 0.05).

## Results

### Mammea E/BB decreased WT1 protein levels and total cell number with increasing time and dose in K562 cells

**Fig. 2:**
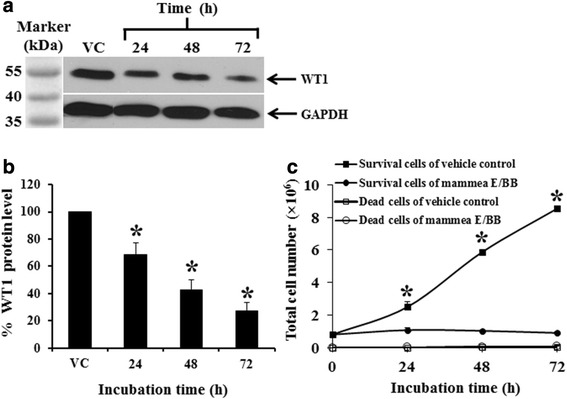
Inhibitory effects of mammea E/BB on WT1 protein levels and total cell number in K562 cells. **a** WT1 protein levels in K562 cells treated with 3.5 μM mammea E/BB for 24, 48, and 72 h, detected by Western blotting. GAPDH was used as a loading control. **b** The protein levels presented as the percentage of vehicle control (0.006 % DMSO alone without the mammea E/BB in the culture medium), analyzed with the scanning densitometer. **c** The total survival and dead cell number of K562 cells after treatment at various times as determined by the trypan blue exclusion method. Data are the mean value ± SEM of three independent experiments. Asterisks (*) denote values that were significantly different from the vehicle control (*p* < 0.05)

The mammea E/BB displayed cytotoxic effects in K562 cells, with an IC_50_ value of 24.1 ± 2.2 μM, and was non-cytotoxic at the IC_20_ value of 3.7 ± 0.2 μM (Additional file 3: Figure S3). The activity of mammea E/BB on WT1 protein expression was examined by Western blotting using a non-cytotoxic dose (3.5 μM). Treatment of K562 cells with mammea E/BB for 24, 48, and 72 h decreased WT1 protein levels as compared to the vehicle control (Fig. 2a and b). Total viable cell numbers at 24, 48, and 72 h were also found to decrease with time. There were no differences in the numbers of dead cells (dead cells accounted for less than 10 %) at each time point between vehicle control and mammea E/BB treatment (Fig. 2c).

**Fig. 3:**
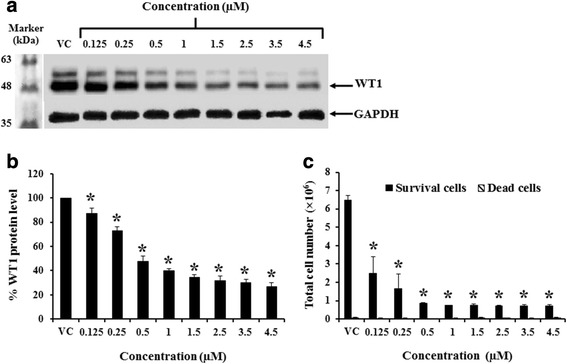
Inhibitory effects of mammea E/BB on WT1 protein levels and total cell number in K562 cells. K562 cells were treated with various concentrations (0.125–4.5 μM) of mammea E/BB for 72 h. **a** WT1 protein levels were detected by Western blotting. GAPDH was used as a loading control. **b** The protein levels presented as the percentage of vehicle control (0.008 % DMSO alone without the mammea E/BB in the culture medium), analyzed with scanning densitometer. **c** The total survival and dead cell number of K562 cells after treatment with various concentrations of mammea E/BB, determined by the trypan blue exclusion method. Data are the mean value ± SEM of three independent experiments. Asterisks (*) denote values that were significantly different from the vehicle control (*p* < 0.05)

The concentrations used for mammea E/BB decreased WT1 protein levels (Fig. 3a and b) and total cell numbers (Fig. 3c).

### Mammea E/BB induced S phase cell cycle arrest in K562

**Fig. 4:**
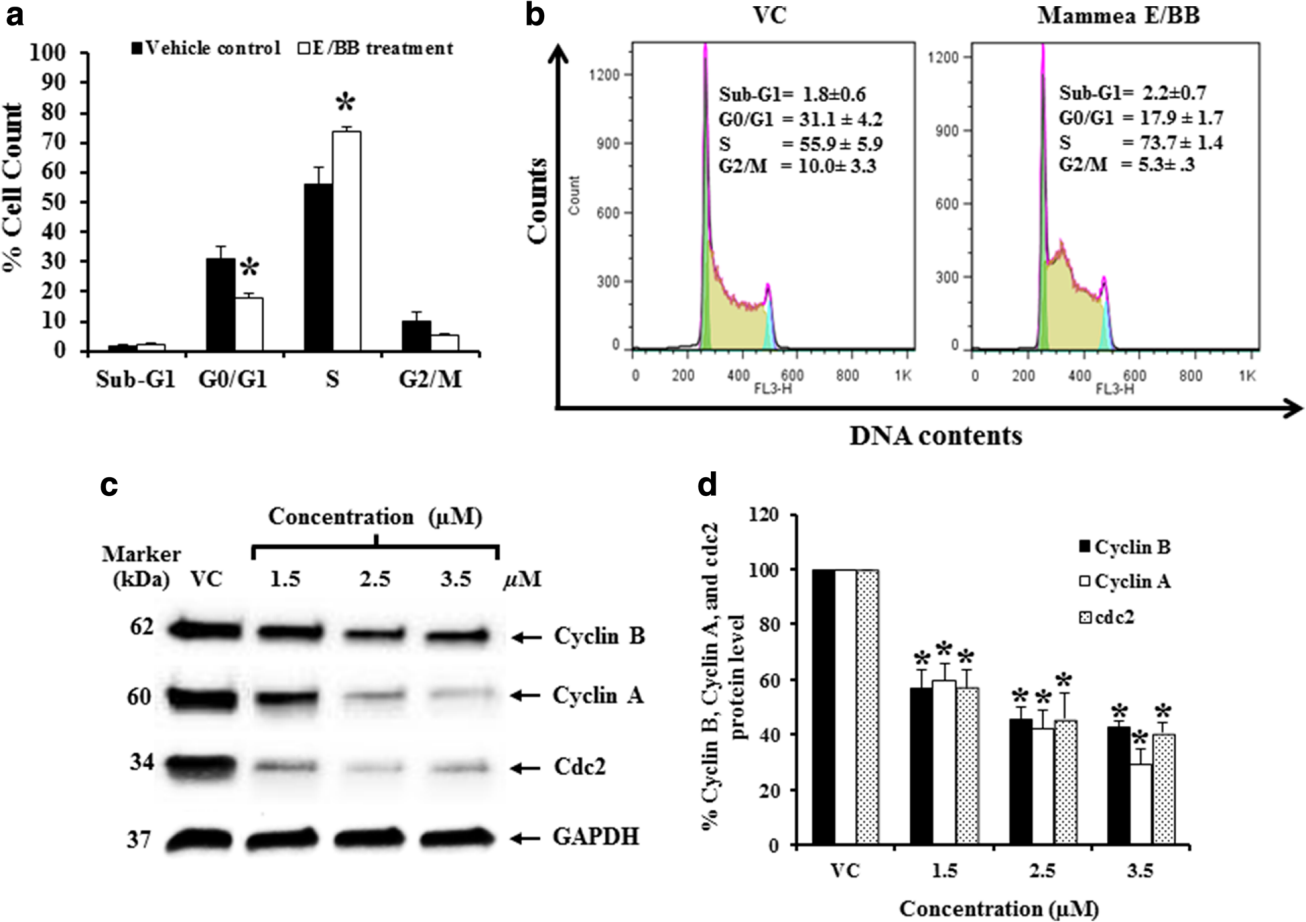
Mammea E/BB treatment induced S phase cell cycle arrest in K562 cells. **a** Cell cycle analysis profile after incubation with 3.5 μmol/L mammea E/BB for 72 h, using flow cytometry. **b** Percentage of sub G1, G0/G1, S, and G2M cell populations of the K562 cells after treatment with mammea E/BB for 72 h are presented and compared to the vehicle control group (VC). **c** Cyclin B, cyclin A, and cdc2 protein levels in K562 cells treated with various concentrations (1.5, 2.5, and 3.5 μM) of mammea E/BB for 72 h, detected by Western blotting. GAPDH was used as a loading control. **d** The protein levels presented as the percentage of vehicle control (0.006 % DMSO alone without the mammea E/BB in the culture medium), analyzed with a scanning densitometer. The percentages of cells in each phase and protein levels are represented as mean ± SEM of three independent experiments. Asterisks (*) denote values that were significantly different from the vehicle control (*p* < 0.05)

Mammea E/BB treatments led to a decline in K562 leukemia cell number without increasing the number of dead cells, suggesting that growth inhibition was mainly through cell cycle arrest. Then, cell cycle arrest was then determined after mammea E/BB treatment by flow cytometry. Indeed, mammea E/BB treatment (3.5 μM) led to a significant accumulation of K562 cells in the S phase of the cell cycle (Fig. 4a and b). Furthermore, cell cycle checkpoint proteins (cyclin A, cyclin B, and cdc2) at the S and G2/M phases were significantly decreased (Fig. 4c and d).

### Effect of mammea E/BB on WT1 protein and WT1 mRNA stability

**Fig. 5:**
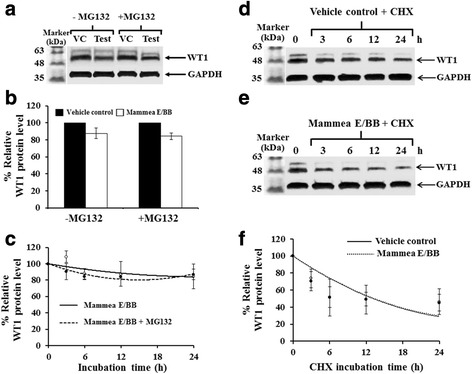
Effect of mammea E/BB on WT1 protein stability. To investigate the effect of mammea E/BB on protein stability, K562 cells were treated with 3.5 μM mammea E/BB in the presence or absence of 5 μM MG132 for 6 h and assessed by immunoblotting. **a** WT1 protein levels from three independent experiments were quantified and are shown in **b**. **c** The effect of 3.5 μM mammea E/BB on WT1 protein levels was examined in the presence or absence of 5 μM MG132 for 0, 3, 6, 12, and 24 h. The relative protein levels were analyzed with a scanning densitometer. The relation of WT1 protein levels to mammea E/BB and mammea E/BB + MG132 were compared to the vehicle control and the MG132 control, respectively. **d**, **e** WT1 protein levels of K562 cells after treated with 50 μg/ml cycloheximide (CHX) in the absence or presence of 3.5 μM mammea E/BB for 0, 3, 6, 12, and 24 h were quantified and are shown in **f**. The relative protein levels were analyzed with a scanning densitometer and compared to the CHX control. GAPDH was used as an internal control. Data are the mean value ± SEM of three independent experiments

**Fig. 6:**
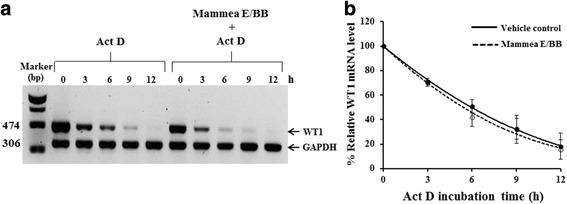
Effect of mammea E/BB on WT1 mRNA stability. To investigate the effect of mammea E/BB on WT1 mRNA stability, K562 cells were treated with 4 μM actinomycin D (Act D) in the presence or absence of 3.5 μM mammea E/BB for 0, 3, 6, 9, and 12 h and WT1 mRNA was assayed by Taqman qRT-PCR as shown in **a**. The WT1 mRNA levels from three independent experiments were quantified and are shown in **b**. GAPDH was used as an internal control. Data are the mean value ± SEM of three independent experiments

To determine whether mammea E/BB has any effect on WT1 protein stability, a time course experiment was performed with the protein synthesis inhibitor, 5 μM MG123 and 50 μg/ml cyclohexamide (CHX). WT1 protein expression decreased after treatment with mammea E/BB in the presence or absence of MG132, indicating that the down-regulation of WT1 protein by mammea E/BB was likely not through proteasomal degradation pathways (Fig. 5a,b, and c). In addition, the WT1 protein’s half-life was examined using the protein synthesis inhibitor, cycloheximide (CHX). The half-life of WT1 protein after mammea E/BB treatment was not significantly different from that of the vehicle control, suggesting that mammea E/BB does not accelerate WT1 protein degradation (Fig. 5d,e, and f). WT1 mRNA stability was then examined using actinomycin D (Act D) treatment in the presence or absence of mammea E/BB. The mammea E/BB treatment did not significantly alter the decay of WT1 mRNA (Fig. 6a and b). Collectively, the results strongly suggest that WT1 repression by mammea E/BB is not through accelerated WT1 protein or mRNA degradation.

### Mammea E/BB attenuates WT1 auto-regulation

**Fig. 7:**
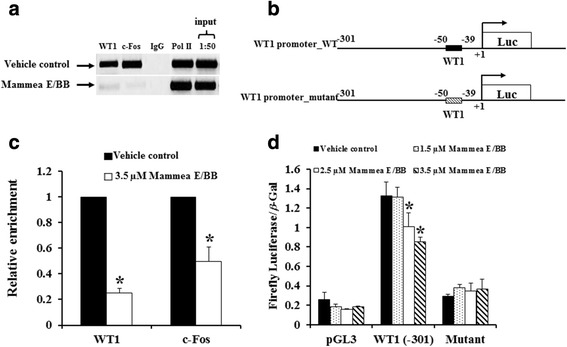
Mammea E/BB treatment attenuated WT1 - DNA binding to the proximal WT1 promoter and WT1 promoter activity. **a** K562 cells were treated with 3.5 μM mammea E/BB for 72 h and ChIPs were performed. Chromatin lysates were immunoprecipitated with antibodies to WT1, c-Fos/AP-1, Pol II (*positive control*) or IgG (*negative control*). ChIP lysates and 1:50 dilution of input were assayed by standard PCR using primers containing the consensus sequence for WT1 located at the WT1 proximal promoter. The WT1 immunoprecipitated lysates from mammea E/BB or vehicle control treatment were analyzed by SYBR green RT-PCR and graphed as relative DNA enrichment over 1:50 input as percentage of vehicle-treatment as shown in **b**. For WT1 promoter reporter activity, K562 cells were transfected with the pGL3_basic luciferase reporter (Luc) vector containing 301 bp of the wild type and mutant WT1 proximal promoter (**c**) followed by 1.5, 2.5, and 3.5 μM or vehicle treatment for 72 h. WT1or a mutant construct was used to transfect for 24 h and then assayed for firefly luciferase and β-galactosidase (β-Gal) activities. Site directed mutagenesis of the WT1 consensus sequence (-50 to -39) abrogated the WT1 promoter activity compared to the wild type WT1 promoter construct (301 bp WT1). The firefly luciferase and β-galactosidase activities were assayed and relative activities were graphed compared to the pGL3 basic vector (**d**). Experiments were performed a minimum of three times and representative graphs are shown. Data are the mean value ± SD of three independent experiments. Asterisks (*) denote values that were significantly different from the vehicle control (*p* < 0.05)

Mammea E/BB was next examined for its impact on the transcriptional regulation of the *WT1* gene using the ChIP assay. WT1 is known to drive its own transcription using an auto-regulatory mechanism. The WT1 promoter has been found to contain one AP-1 consensus sequence, TGAGTGA, at +144 to +150. Treatment of K562 cells with 3.5 μM mammea E/BB for 72 h could significantly inhibit WT1 binding to its own promoter, by up to 75 % (Fig. 7a and b). Mammea E/BB also disrupted c-Fos/AP-1 binding to the WT1 promoter by 50 % as compared to the vehicle control by standard PCR.

The minimal promoter element essential for *WT1* gene expression is the WT1 proximal promoter (-301 bp) [20]. The WT1 (-50 to -39) consensus binding site is included in this proximal promoter element (Fig. 7c). Transfection of this 301 bp construct, contained within the pGL3 reporter vector into K562 cells, demonstrated high luciferase activity in vehicle control treated cells and a diminished response with the various concentrations of mammea E/BB (1.5, 2.5, and 3.5 μM) treated K562 cells (Fig. 7d). As a control, a reporter construct containing a mutated WT1 binding site was included. The mutant vector did not show significantly increased activity compared to the empty pGL3 vector, demonstrating that this assay is mostly driven by WT1 binding.

### Mammea E/BB decreases p-ERK1/2 (Thr202/Tyr204) in K562 cells

**Fig. 8:**
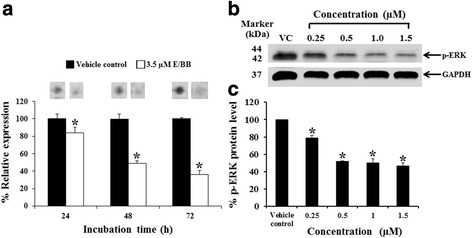
Effect of mammea E/BB on p-ERK1/2 in K562 cells. **a** K562 cells were treated with 3.5 μM mammea E/BB for 72 h using a human phospho-kinase array kit. Cells were treated with various concentrations for 72 h. **b** The p-ERK1/2 protein levels of K562 cells were detected by Western blotting. The levels of WT1 protein expression were assessed by immunoblotting; GAPDH was used as a loading control. **c** The protein levels were analyzed with a scan densitometer and the graphs were plotted as the percentage of vehicle control (0.003 % DMSO alone without the mammea E/BB in the culture medium). Data are the mean value ± SEM of three independent experiments. Asterisks (*) denote values that were significantly different from the vehicle control (*p* < 0.05)

To gain insight into the impact of mammea E/BB on cellular signaling, a kinase signaling array was used to examine the phosphorylation/activation state of various key kinases. In this experiment, K562 cells were treated with non-cytotoxic doses of mammea E/BB (3.5 μM) for 72 h. As shown in Fig. 8, mammea E/BB strongly decreased p-ERK1/2 (Thr202/Tyr204) in K562 cells (Fig. 8a). To confirm the signaling array results, p-ERK1/2 phosphorylation was examined with conventional Western blotting following mammea E/BB treatment of K562 cells (Fig. 8b and c). Mammea E/BB treatment led to a significant decrease in ERK1/2 phosphorylation.

## Discussion

To date, saraphi (*M. siamensis*) has been used in Thai folk medicine remedies with limited scientific knowledge, especially regarding its biological activity in leukemic cells. In this study, mammea E/BB (the active compound in *M. siamensis* seeds) was examined for its down-regulatory mechanism to suppress leukemic cell proliferation via inhibiting WT1 protein expression. Mammea E/BB has been reported to impact cancer cells [28]. It is an important active compound in the nonpolar part of *M. siamensis* seed extract. The results of the present study demonstrate that mammea E/BB inhibited cell proliferation and repressed WT1 expression in the human leukemia K562 cell line.

The activities of mammea E/BB on WT1 protein expression and total viable cell number were found to decrease with increasing time and dose by Western blotting and trypan blue exclusion method, respectively. There was no difference in the number of dead cells (dead cells accounting for less than 10 %) at each time point between the vehicle control and mammea E/BB treatment.

The effect of various concentrations of mammea E/BB was examined and found to decrease WT1 protein levels in response to 0.125 to 4.5 μM by Western blotting. Moreover, the total cell number was significantly decreased at 72 h by 61.3 ± 8.9 to 89.2 ± 0.8 %, as compared to the vehicle control. The dead cells accounted for less than 10 %. Mammea E/BB treatments led to a decline in K562 leukemia cell number without increasing the number of dead cells, suggesting that growth inhibition is mainly through cell cycle arrest. Thus, the effect of mammea E/BB on cell cycle progression was investigated. Indeed, mammea E/BB treatment (3.5 μM) led to a significant decline in K562 cells in the S phase of the cell cycle as assessed by flow cytometry. Furthermore, the proteins related to S and G2/M phase control were determined. It was found that cell cycle checkpoint proteins (cyclin A, cyclin B, and cdc2) at the S and G2/M phases were significantly decreased in a dose-dependent manner.

This study next examined whether WT1 down-regulation by mammea E/BB was through effects on protein or mRNA degradation pathways. K562 cells occurred treated with 3.5 μM mammea E/BB (IC_20_) for 24 h in the presence or absence of the proteasome inhibitor MG132. WT1 protein expression decreased similarly after treatment with mammea E/BB in the presence or absence of MG132, indicating that mammea E/BB-mediated down-regulation of WT1 protein was likely not through proteasomal degradation pathways. In addition, WT1 protein half-life was examined using the protein synthesis inhibitor, cycloheximide (CHX). The half-life of WT1 protein after mammea E/BB treatment was not significantly different as compared to the vehicle control, suggesting that mammea E/BB does not accelerate WT1 protein degradation. WT1 mRNA stability was examined using actinomycin D (Act D) treatment in the presence or absence of mammea E/BB. The results demonstrated that mammea E/BB treatment did not significantly alter the decay of WT1 mRNA. Collectively, the results strongly suggest that WT1 repression by mammea E/BB is not through accelerated WT1 protein or mRNA degradation but rather is related to the WT1 signaling pathway.

The experiment further examined whether mammea E/BB impacts the transcriptional regulation in signal transduction of the *WT1* gene using the ChIP assay. It was found that treatment of K562 cells with mammea E/BB for 72 h could significantly inhibit WT1 binding to its own promoter by 75 %. WT1 is known to drive its own transcription using an auto-regulatory mechanism. Mammea E/BB also disrupted c-Fos binding to the WT1 promoter. C-Fos/AP-1 is the heterodimeric partner of c-Jun, together creating the AP-1 transcription factor. AP-1 is known to play an important role in cell proliferation [29] and the WT1 promoter has one AP-1 consensus sequence, TGAGTGA, at +144 to +150. C-Fos/AP-1 must be phosphorylated by MAPK/ERK in order to function as a transcription factor [30] and mammea E/BB may inhibit c-Fos/AP-1 recruitment to the WT promoter by inhibiting ERK1/2 phosphorylation (discussed below).

The minimal promoter element that is essential for *WT1* gene expression is the WT1 proximal promoter (-301 bp) [20]. The WT1 consensus binding site (-50 to -39) is included in this proximal promoter element. Transfection of this 301 bp construct, contained within the pGL3 reporter vector into K562 cells demonstrated high luciferase activity with vehicle control treated cells and a diminished response with the various concentrations of mammea E/BB (1.5, 2.5, and 3.5 μM). As a control, a reporter construct containing a mutated WT1 binding site was included. The mutant vector did not show significantly increased activity compared to the empty pGL3 vector, demonstrating that this assay is mostly driven by WT1 binding.

Mammea E/BB had varying effects on WT1 signaling in K562 cells and led to a significant decrease in p-ERK1/2 phosphorylation. Given the wide-ranging roles for ERK1/2 in cell proliferation and survival, inhibition of ERK1/2 phosphorylation may contribute to mammea E/BB’s biological activity. These findings demonstrate that mammea E/BB decreased WT1 protein expression and induced S phase cell cycle arrest in leukemia cells. This study uses an in vitro experimental model and will be further studied in an animal models and clinical trials. In the previous study, WT1 expression in patient leukemic cells was determined after turmeric curcumin treatment and WT1 mRNA was decreased after treatment, especially in patients with a high level of *WT1* gene expression patients [31].

## Conclusion

The current study has demonstrated that pure mammea E/BB-mediated down-regulation of WT1 was not the result of protein or mRNA degradation processes. Rather, both chromatin immunoprecipitation (ChIP) and luciferase reporter assays indicate that mammea E/BB interferes with WT1 and c-Fos/AP-1 binding to their DNA consensus sites at the proximal promoter of the *WT1* gene, nullifying WT1’s positive auto-regulatory role. Therefore, a low dose of mammea E/BB functions to inhibit WT1 expression at the transcriptional regulatory level and induce S phase cell cycle arrest in K562 cells. This novel mechanistic knowledge of how mammea E/BB affects WT1 transcriptional function in leukemic cells may be useful in the future development of the natural product for therapeutic treatment of leukemia patients.

## Ethics approval and consent to participate

Not applicable.

## Consent for publication

Not applicable.

## Availability of data and materials

The datasets supporting the conclusions of this article are included within the article and additional files.

## Additional files

Additional file 1: Figure S1.
^1^H NMR spectrum (400 MHz, CDCl_3_) of mammea E/BB performed on a Bruker AVANCE 400 NMR spectrometer. (TIF 132 kb) Additional file 2: Figure S2.
^13^C NMR spectrum (101 MHz, CDCl_3_) of mammea E/BB performed on a Bruker AVANCE 400 NMR spectrometer. (TIF 136 kb) Additional file 3: Figure S3. Cytotoxic effect of mammea E/BB at 72 h on K562 cell line by the MTT assay. Each point represents the mean ± SEM of three independent experiments performed in triplicate. (TIF 27 kb)
