# Supplementary figures and images for: Down-regulatory mechanism of mammea E/BB from Mammea siamensis seed extract on Wilms’ Tumor 1 expression in K562 cells
Source: BMC Complement Altern Med. 2016 May 18;16:130. doi: 10.1186/s12906-016-1107-z (PMC4870773; doi:10.1186/s12906-016-1107-z)

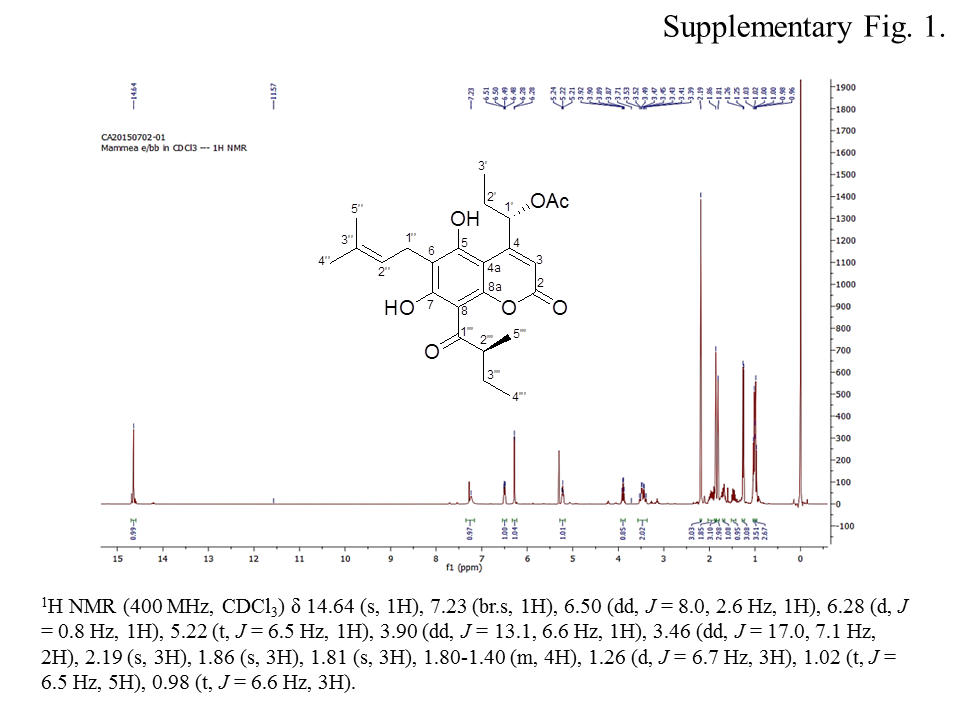

Supplement: Additional file 1: Figure S1. — 1H NMR spectrum (400 MHz, CDCl3) of mammea E/BB performed on a Bruker AVANCE 400 NMR spectrometer. (TIF 132 kb) [file 12906_2016_1107_MOESM1_ESM.tif]

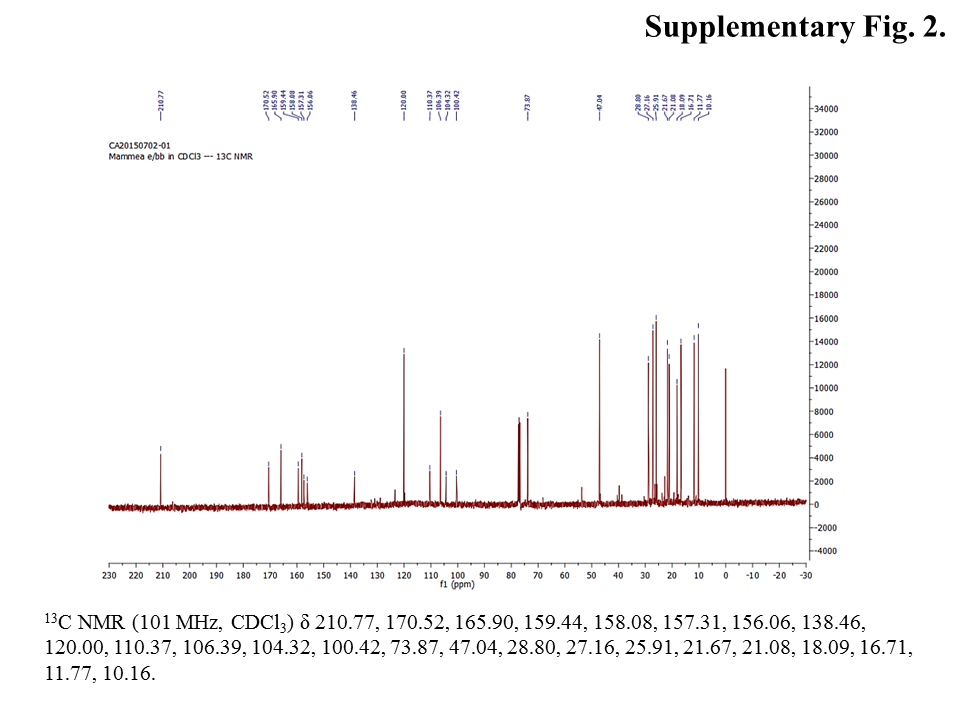

Supplement: Additional file 2: Figure S2. — 13C NMR spectrum (101 MHz, CDCl3) of mammea E/BB performed on a Bruker AVANCE 400 NMR spectrometer. (TIF 136 kb) [file 12906_2016_1107_MOESM2_ESM.tif]

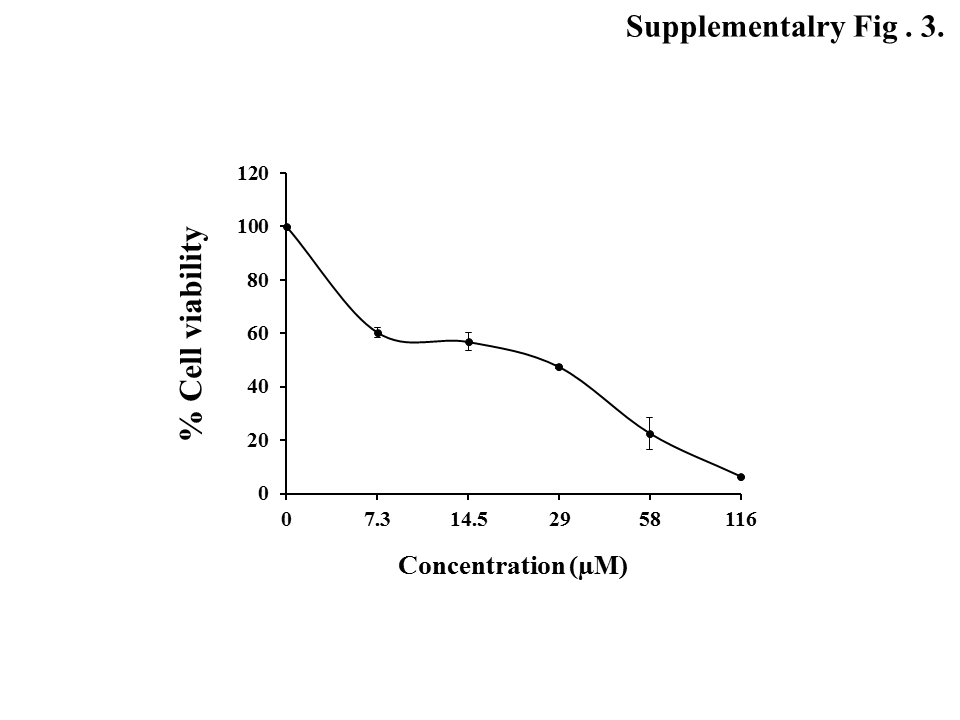

Supplement: Additional file 3: Figure S3. — Cytotoxic effect of mammea E/BB at 72 h on K562 cell line by the MTT assay. Each point represents the mean ± SEM of three independent experiments performed in triplicate. (TIF 27 kb) [file 12906_2016_1107_MOESM3_ESM.tif]
